# Partitionable High-Efficiency Multilayer Diffractive Optical Neural Network

**DOI:** 10.3390/s22197110

**Published:** 2022-09-20

**Authors:** Yongji Long, Zirong Wang, Bin He, Ting Nie, Xingxiang Zhang, Tianjiao Fu

**Affiliations:** 1 Changchun Institute of Optics, Fine Mechanics and Physics, Chinese Academy of Sciences, Changchun 130033, China; 2 University of Chinese Academy of Sciences, Beijing 100049, China

**Keywords:** optical neural network, diffraction, optical computing

## Abstract

A partitionable adaptive multilayer diffractive optical neural network is constructed to address setup issues in multilayer diffractive optical neural network systems and the difficulty of flexibly changing the number of layers and input data size. When the diffractive devices are partitioned properly, a multilayer diffractive optical neural network can be constructed quickly and flexibly without readjusting the optical path, and the number of optical devices, which increases linearly with the number of network layers, can be avoided while preventing the energy loss during propagation where the beam energy decays exponentially with the number of layers. This architecture can be extended to construct distinct optical neural networks for different diffraction devices in various spectral bands. The accuracy values of 89.1% and 81.0% are experimentally evaluated for MNIST database and MNIST fashion database and show that the classification performance of the proposed optical neural network reaches state-of-the-art levels.

## 1. Introduction

Deep learning is a machine learning method that predicts data by simulating multilayer artificial neural networks. Deep learning is widely used in various fields, including medicine [1,2], communication [3,4], security [5,6], computer vision [7], and the military. With the rapidly increasing demands for artificial neural network applications, the computation and performance requirements have increased dramatically, and the development of existing neural networks has faced challenges due to bottlenecks in the development of traditional silicon-based chips in the following two aspects. On the one hand, the von Neumann architecture has difficulty satisfying the needs of large-scale neural network computing; on the other hand, silicon-based chips have difficulty satisfying the needs of large-scale neural network computing due to power consumption and heat issues, which limit the clock frequency; thus, it is difficult to enhance the performance of single-core systems and the computing power efficiency ratio. At present, according to the low complexity and high data volume characteristics of neural network computations, several commercial companies [8] have increased the number of computing cores in silicon-based chips to meet the considerable computational demands of large-scale neural networks; however, this method does not fundamentally address the bottleneck problem faced by silicon-based chips in neural network computations, and the increase in the number of cores is not linear with improvements in computational performance; therefore, bypassing silicon-based chips and instead using optical computing to build artificial neural networks has become a new focus in neural network research.

The use of optical systems to implement Fourier transform, correlation and convolution operations has long been valued by researchers [9] because optical computing has the advantages of low power consumption, high parallelism, and fast speeds, and can thus satisfy the needs of massive data processing. In recent years, as a result of the development of optoelectronic devices, optical computing research is no longer limited to the computation of Fourier transforms [10] and has been applied to the construction of artificial neural networks. The diffractive optical neural network (D2NN) was proposed by Ozcan et al. [11,12,13,14]. Based on the error back-propagation method, D2NN uses computer training to obtain the phase distribution of each diffractive optics element layer. During the training process, each pixel of the diffractive optics element layer is a neuron, and the computer optimizes the phase of each pixel of the diffractive optics element layer by constraining the light field distribution after passing through the diffractive optics element layer. After training, the phase of each pixel in the diffractive optics layer is printed as a phase mask by a 3D printer. The input to the diffractive optical neural network is achieved by shining a terahertz light source onto an aluminum foil etched with the input image information. The network output is scanned point by point in the output plane by a single pixel detector. D2NNs working in the terahertz band demonstrated high parallelism in optical computing, but because of the terahertz wavelength, the size of D2NNs is limited, making their processing and application more difficult. Chen et al. [15] experimentally verified the D2NN operating in the visible band and proposed a revised formula for neuron size and wavelength for the visible band. The phase mask for the visible band in Chen’s experiments was fabricated by etching a quartz substrate, and the output of the diffractive optical neural network was captured directly by a CCD detector for the light intensity distribution in the output plane. The application of D2NN to visible wavelengths reduces the size of diffractive optical neural networks and makes the application of diffractive optical neural networks further a reality, but the lack of nonlinear activation functions compared to conventional electrical neural networks limits the performance of optical diffractive neural networks. To implement the nonlinear activation function in optical neural networks, Zou et al. [16] used the nonlinear optical properties of two-dimensional magneto-optical traps to implement optical activation functions. Li et al. [17] used the response of optoelectronic imaging devices to implement the activation function in an optical neural networks. In addition, Zhou et al. [18] used a four-step phase-shifted digital holography technique to collect the middle-layer light field in real time during training and fed the light field back into the network during training to correct errors between the actual optical path and the simulation model, improving the robustness of the model and reducing the difficulty of optical experiments. Furthermore, they implemented deep neural networks and recurrent neural networks [19] and used photodetectors to collect the light field, as well as multiple spatial light modulators for transmission.

Although there have been many excellent research studies on diffractive optical neural networks [10,11,12,13,14,16,17,18,19,20], the application of these research results in engineering remains difficult. The experiments of Chen et al. [15] require precise alignment of multiple quartz phase masks, and the experiments of Zhou et al. [18] require precise measurement of the optical field using a four-step phase shift method. In addition, the modulation rate of the optical modulation device and the acquisition rate of the photodetector device limit the practical applications of optical neural networks; therefore, existing diffraction optical neural networks should be improved. For example, the robustness of the mechanical mounting error in the optical neural network can be improved to reduce the accuracy requirement of mounting the optical neural network, thus reducing the impact of temperature changes or vibrations on the system in practical application environments. Moreover, parallel input and output methods can be used to increase the computational speed of optical neural networks, which is limited due to the insufficient refresh rate of existing photoelectric modulation devices.

Furthermore, the optical neural network should use a reasonable optical design to adaptively adjust to the size of the input and output data, thus improving the computational efficiency and speed.

In this paper, we propose partitioning a multilayer optical neural network in planar space optical modulation device and photodetector device.

This method addresses the shortcomings of previous multilayer diffractive optical neural networks, which face difficulties in flexibly changing the number of layers in the network and the size of the input data. This system can improve the computational efficiency of the diffractive optical neural network while reducing the number of optical devices and the difficulty in aligning the optical path. In addition, holograms are introduced to assist in calibrating the positions of the phase plate and output plane, and the nonlinear characteristics of the photodetector are used to realize a nonlinear activation function in the optical neural network.

## 2. Principle and Analysis

### 2.1. Optical Neural Network Based on Fresnel–Kirchhoff Diffraction

**Figure 1:**
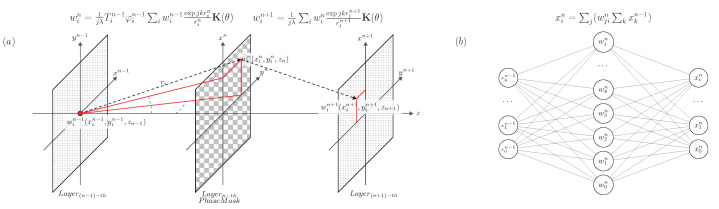
(**a**) Propagation model of the diffractive neural network layer. (**b**) Single fully connected layer in the digital neural network model.

The model of the conventional digital fully connected neural network layer is shown in Figure 1b, where {$x_{0}^{n-1}$, $x_{1}^{n-1}$, ⋯, $x_{k}^{n-1}$} are the input layer data, {$x_{0}^{n}$, $x_{1}^{n}$, ⋯, $x_{i}^{n}$} are the output layer data, and {$w_{0}^{n}$, $w_{1}^{n}$, ⋯, $w_{j}^{n}$} are the hidden layer weight values. Thus, the fully connected neural network layer can be written as:

(1) $$\begin{array}{cc} \; & x_{i}^{n}=\sum_{j}\left(w_{ji}^{n}\sum_{k}x_{k}^{n-1}\right) \end{array}$$

The output $x^{n}$ of a simple neural network layer is the sum of the products of the input data $x^{n-1}$ and the corresponding weight values $w^{n}$. In the field of optics, according to Huygens’ principle, Fresnel–Kirchhoff diffraction can be expressed as subwaves being emitted from each point of the wavefront; these subwaves interfere with each other and superimpose to form a new wavefront [21]. The calculation of the Fresnel–Kirchhoff diffraction for the discrete case is shown in Figure 1a. $Layer_{n-th}$ is the phase plane, and the phase distribution in the $Layer_{n-th}$ phase plane can be denoted as $\varphi^{n}\left(x^{n},y^{n},z_{n}\right)$. The transmittance is $T^{n}\left(x^{n},y^{n},z_{n}\right)$. The wavefront $w^{n-1}\left(x^{n-1},y^{n-1},z_{n-1}\right)$ after the wavefront $w^{n-1}\left(x^{n-1},y^{n-1},z_{n-1}\right)$ from the point source in the $Layer_{(n-1)-th}$ plane passes through the $Layer_{n-th}^{n}\left(x^{n},y^{n},z_{n}\right)$ phase plane is:

(2) $$\begin{array}{cc} \; & w^{n}=T^{n}\exp\left(j\varphi^{n}\right)t\left(w^{n-1}\right)\\ \; & t\left(w^{n-1}\right)=\frac{1}{j\lambda }\sum_{i}w_{i}^{n-1}\frac{\exp jkr_{i}^{n-1}}{r_{i}^{n-1}}K\left(\theta \right)\\ \; & r_{i}=\sqrt{{\left(x^{n}-x_{i}^{n-1}\right)}^{2}+{\left(y^{n}-y_{i}^{n-1}\right)}^{2}+{\left(z_{n}-z_{n-1}\right)}^{2}}\\ \; & k=\frac{2\pi }{\lambda }\\ \; & K\left(\theta \right)=\frac{\left(z_{n}-z_{n-1}\right)}{r_{i}}\\ \; & T\in (0,1),\varphi \in [0,2\pi ] \end{array}$$

where λ is the wavelength, θ is the angle between $\vec{r}$ and the normal vector $\vec{z}$ of the $Layer_{(n-1)-\mathrm{th}}$ plane, and $r_{i}$ is the optical path of the light ray passing from point $\left(x^{n-1},y^{n-1},z_{n-1}\right)$ in the $Layer_{(n-1)-\mathrm{th}}$ plane to point $\left(x^{n},y^{n},z_{n}\right)$ in the $Layer_{n-\mathrm{th}}$ plane.

The optical model shown in Figure 1a is a model of the optical neural network layer, where the wavefront $w^{n-1}\left(x^{n-1},y^{n-1},z_{n-1}\right)$ in the $Layer_{(n-1)-\mathrm{th}}$ plane is the input data of the neural network layer, the phase distribution $\varphi^{n}\left(x^{n},y^{n},z_{n}\right)$ in the $Layer_{n-\mathrm{th}}$ phase plane is the weight value of the hidden layer, and the wavefront $w^{n+1}\left(x^{n+1},y^{n+1},z_{n+1}\right)$ in the $Layer_{(n+1)-\mathrm{th}}$ plane is the output data of the neural network layer.

A digital neural network model usually includes multiple network layers to enhance the expression ability of the model, and the corresponding diffractive optical neural network can realize a deep neural network with *n* layers of *n* diffractive optical systems in series. According to Equation (2), the n-layer deep network composed of *n* diffractive optical systems in series can be described by the following formula:

(3) $$\begin{array}{cc} \; & w^{n}=F^{n}\left(w^{0},T,\varphi \right)\\ \; & F^{n}=f\left(w^{n-1},T^{n},\varphi^{n}\right)\\ \; & =f(f\left(w^{n-2},T^{n-1},\varphi^{n-1}\right),T^{n},\varphi^{n})\\ \; & =f(f\left(\cdots f\left(w^{0},T^{1},\varphi^{1}\right),\cdots \right),T^{n},\varphi^{n})\\ \; & f\left(w^{0},T^{1},\varphi^{1}\right)=T^{1}\varphi^{1}\frac{1}{j\lambda }\sum_{i}w_{i}^{0}\frac{\exp jkr_{i}^{0}}{r_{i}^{0}}K\left(\theta \right)\\ \; & E\left(\varphi \right)={\left(\left(w^{n}\right)*\cdot w^{n}-G\right)}^{2}\\ \; & \min_{\varphi }E\left(\varphi \right),\;s\cdot t\;T\in \left(0,1\right),\varphi \in \left[0,2\pi \right] \end{array}$$

where $F^{n}$ is the transfer function of the *n*-layer diffractive optical neural network composed of *n* diffractive optical systems in series, *f* is the transfer function of the diffractive optical system, and *G* is the expected output optical field of a diffractive optical system with an input optical field of $w^{0}$. Corresponding to the digital neural network model, $w^{0}$ is the input of the model, $w^{n}$ is the output of the model, and *T* and φ are the weights of the model.

### 2.2. Multilayer Diffractive Optical Neural Network with Partitioned Multiplexing

**Figure 2:**
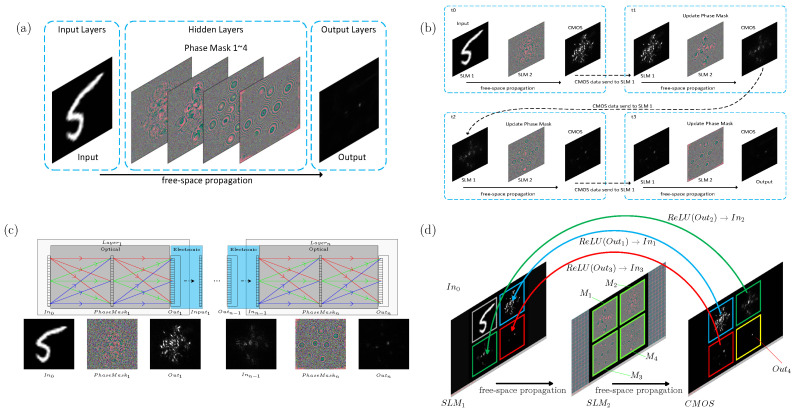
(**a**) Typical diffractive optical neural network architecture. (**b**) Four-layer optical neural network implemented by a single optical hybrid neural network layer unit. (**c**) Diffractive optical neural network composed of multiple optoelectronic hybrid neural network layers ($In_{0}$: input data of the network, $PhaseMark_{n}$: weights of the *n*th layer, $Out_{n}$: output of the network). (**d**) Four-layer diffractive optical neural network implemented by partitioning the optoelectronic hybrid neural network layers.

A typical all-optical diffraction neural network model is shown in Figure 2a, where the optical field information of the input plane *Input* is the input layer data, the optical field information of the output plane *Output* is the output layer data, a diffraction layer with multiple phase plates is the hidden layer, and the phase delay of the wavefront passing through the phase plates is the weight value of the hidden layer. Although the all-optical diffraction neural network shown in Figure 2a can be implemented as a deep neural network by simply increasing the number of diffraction layers without increasing the power consumption of the system, it is challenging to flexibly change the number of phase plates in an optical system. To address the challenge of flexibly changing the structure and number of layers in an optical diffraction neural network, we propose a hybrid optical neural network. Figure 2b shows a hybrid optical diffraction neural network with four hidden layers and the computation process of this hybrid network. The layers of the hybrid optical neural network with nonlinear activation functions follow the process shown in Figure 2b. First, the computation of the current layer is used to obtain the output of the current layer, which is used as the input of the next layer; then, the weights of the phase plane are updated as the weights of the next layer. The computation of the next layer in the network follows the same process. The data input to the optical hybrid neural network layer is realized by an amplitude-only spatial light modulator (SLM 1), the phase plane of the diffraction layer is realized by a phase-only spatial light modulator (SLM 2), and the data output is obtained by CMOS acquisition of the intensity distribution of the light field. The nonlinear activation function is realized by using a photoelectric conversion device to acquire the light field intensity distribution in the output plane after the diffraction layer. The process of the nonlinear activation function is as follows: the photodetector acquires the light field intensity distribution after the diffraction layer, passes the data through the nonlinear activation function, and then transmits the data to the amplitude-only spatial light modulator.

Figure 2c shows a multilayer neural network model composed of multiple photoelectric hybrid optical diffraction neural network layers. The white box $Layer_{1}$ in the figure is the optical diffraction neural network layer, $In_{0}$ is the input surface, $PhaseMask_{1}$ is the phase surface, and $Out_{1}$ is the output surface. The diffractive optical system composed of *n* optical diffractive neural network layers in series is an *n*-layer deep optical neural network that can be described by Equation (3).

In this formula, the input $w^{0}$ is the light field at the input surface $In_{0}$ of the 1st network layer $Layer_{1}$, the weight $\varphi^{1}$ is the phase at the phase plane $PhaseMask_{1}$ of the 1st network layer $Layer_{1}$, and $w^{1}$ is the light field at the output surface $Out_{1}$ of the 1st layer $Layer_{1}$.

The weight $\varphi^{n}$ is the phase at the $PhaseMask_{n}$ phase plane in the *n*-th network layer $Layer_{n}$. The output $w^{n}$ of the network is the light field intensity at the output surface $Out_{n}$ of the *n*-th network layer $Layer_{n}$.

Although the optical neural network shown in Figure 2c improves the computational efficiency by computing multiple network layers in a pipeline with several optoelectronic hybrid neural network layers, the system complexity also increases.

Thus, the optical adjustment accuracy of the multiple network layers should be ensured, as a large number of optoelectronic components may lead to an increase in power consumption.

The proposed multilayer optical diffraction neural network model is shown in Figure 2d. This model uses one amplitude-only spatial light modulator (SLM 1), one phase-only spatial light modulator (SLM 2), and one photodetector (CMOS) to realize parallel pipeline computations in the multilayer optical diffraction neural network. The model in Figure 2d implements pipeline computations in a four-layer optical diffraction neural network. The four regions $In_{0,1,2,3}$ in SLM 1 are the input planes of the 1st through 4th network layers, $w_{in}^{0,1,2,3}$ are the optical fields at the input planes $In_{0,1,2,3}$, and the four regions $M_{1,1,2,3}$ in SLM 2 are the optical fields at the input planes $M_{1,1,2,3}$. The four regions $M_{1,2,3,4}$ are the phase planes of the 1st through 4th network layers, and $\varphi^{0,1,2,3}$ are the phases of the phase planes $M_{0,1,2,3}$. The four regions $Out_{1,2,3,4}$ are the output planes of the 1st through 4th network layers, and $w_{Out}^{1,2,3,4}$ are the optical fields at the output surface $Out_{1,2,3,4}$. The 4-layer diffraction neural network model shown in Figure 2d can be calculated with Equation (4).

(4) $$\begin{array}{cc} \; & w_{Out}^{4}=t_{2}\left(exp\left(j\varphi^{4}\right)t_{1}\left(w_{in}^{3}\right)\right)T\\ \; & w_{Out}^{3}=t_{2}\left(exp\left(j\varphi^{3}\right)t_{1}\left(w_{in}^{2}\right)\right)T\\ \; & w_{Out}^{2}=t_{2}\left(exp\left(j\varphi^{2}\right)t_{1}\left(w_{in}^{1}\right)\right)T\\ \; & w_{Out}^{1}=t_{2}\left(exp\left(j\varphi^{1}\right)t_{1}\left(w_{in}^{0}\right)\right)T\\ \; & w_{in}^{i}=ReLU\left({w_{Out}^{i}}^{*}\cdot w_{Out}^{i}\right),i=1,2,3\\ \; & t_{1}\left(w\right)=\frac{1}{j\lambda }\sum_{i}^{n}w_{i}\frac{\exp jkr_{i}^{1}}{r_{i}^{1}}K\left(\theta \right)\\ \; & t_{2}\left(w\right)=\frac{1}{j\lambda }\sum_{i}^{n}w_{i}\frac{\exp jkr_{i}^{2}}{r_{i}^{2}}K\left(\theta \right)\\ \; & E\left(\varphi \right)={\left({w_{Out}^{4}}^{*}\cdot w_{Out}^{4}-G\right)}^{2}\\ \; & \min_{\varphi }E\left(\varphi \right),\;s\cdot t\;\varphi \in \left[0,2\pi \right] \end{array}$$

where $t_{1}\left(w\right)$ is the diffraction equation of the wavefront *w* from the plane region at SLM 1 to the plane region at SLM 2, $r_{i}^{1}$ is the optical path of the secondary wave $w_{i}$ from the wavefront *w* at the plane region at SLM 1 to the plane region at SLM 2, $t_{2}\left(w\right)$ is the diffraction equation of the wavefront *w* from the plane region at SLM 2 to the plane region at the CMOS photodetector, and $r_{i}^{2}$ is the optical path of the secondary wave $w_{i}$ from the wavefront *w* at the plane region at SLM 2 to the plane region at the CMOS photodetector. $w_{in}^{0}$ is the input data to the diffractive optical neural network, and *G* is the input data label corresponding to the optical field distribution. *T* is transmittance of the optical systems. ReLU is the nonlinear activation function, which is obtained according to the CMOS optical conversion characteristics and can be written as Equation (5):

(5) $$\begin{array}{c} ReLU\left(x\right)=\left\{\begin{array}{cc} Max, & x>Max\\ x, & Max>x>Min\\ 0, & x<Min \end{array}\right. \end{array}$$

where Max is the maximum unsaturated light intensity detectable by CMOS and Min is the activation threshold of the ReLU activation function. Min is greater than the minimum light intensity detectable by CMOS.

## 3. Experiments

**Figure 3:**
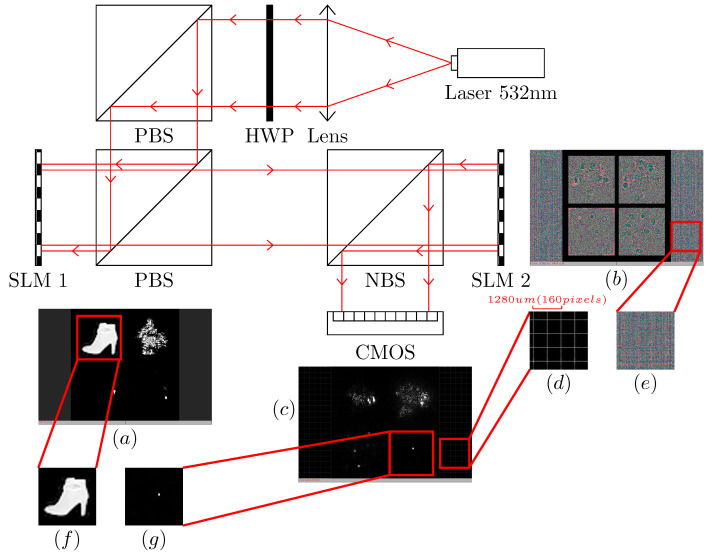
PBS: polarizing beamsplitter cube; NBS: nonpolarizing beamsplitter cube; SLM: spatial light modulator (amplitude/phase); HWP: half-wave plate. (**a**) Input of SLM 1, (**b**) phase mask of SLM 2, (**c**) CMOS capture, (**d**) hologram pattern for alignment, (**e**) phase mask of hologram for alignment, (**f**) input to the neural network, and (**g**) output of the neural network.

The optical experimental verification system of the proposed partitionable optoelectronic hybrid diffraction optical neural network is shown in Figure 3. The system uses a 532 nm polarized coherent laser source (Changchun New Industries Optoelectronics MGL-III-532-100 mW). The expanded laser is adjusted to an *S*-polarized beam by a half-wave plate (Daheng GCL-060633), and the beam is incident on an amplitude-only spatial light modulator, which we denote as SLM 1 (UPOLabs HDSLM80R). SLM 2 (UPOLabs HDSLM80R Plus) is a phase-only spatial light modulator that is used to load the phase plane weights. The CMOS photodetector (Daheng MER2-2000-19U3M-L) acquires the intensity distribution of the light field modulated by the phase mask in the output plane. SLMs 1 and 2 have a resolution of 1920×1200 pixels, with a pixel size of 8 μm, and the SLMs operate in 8 bit mode. The CMOS resolution is 5496×3672 pixels, with a pixel size of 2.4 μm, and the image element sampling depths are 8 bits and 12 bits. The training computer configuration is as follows: the CPU is an Intel Core i7 10700, the GPU is a NVIDIA RTX 3090 ×2 with 64 G of RAM, Windows 11, Python 3.8, and TensorFlow 2.6 with CUDA 11.3.

### 3.1. Experimental Design and Setup

**Figure 4:**
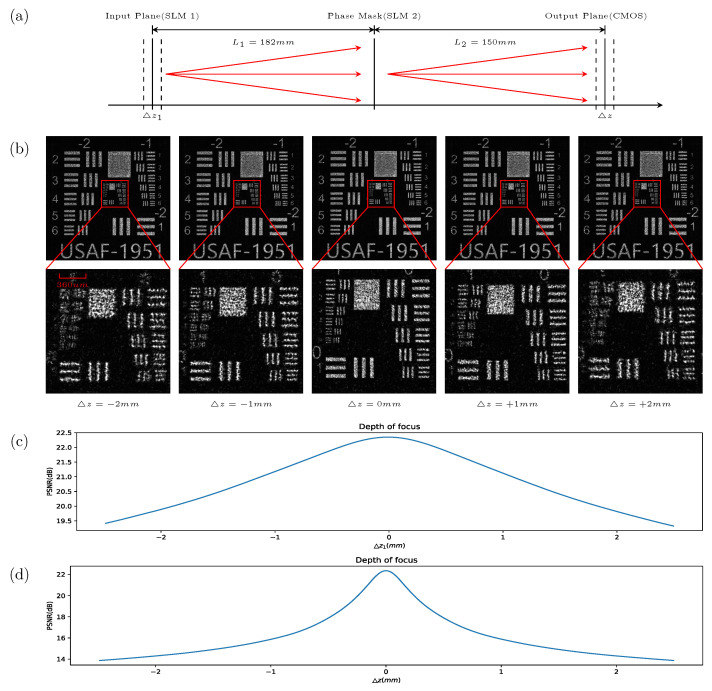
The effect of distance on the hologram pattern. (**a**) Experimental setup to determine the effect of the distance on the hologram pattern. (**b**) The effect of the hologram pattern with displacements of ±1mm or ±2mm in the optical axis direction. (**c**) The effect of the input plane displacement on the hologram pattern in the output plane. (**d**) The effect of the output plane displacement on the hologram pattern in the output plane.

To ensure that the neuron nodes in the optical neural network are linked correctly, the positions of the main optical surfaces in the optical system shown in Figure 3 need to be determined. In this paper, holograms are used as a reference to align the spatial light modulators (SLM 1 and SLM 2) with CMOS. According to Equation (2), the phase distribution φ in the SLM 2 phase plane can be calculated by using the USAF-1951 resolution test pattern as the wavefronts $w^{n-1}$ and $w^{n}$ in the SLM 1 input plane and the CMOS output plane. The effects of the input plane, phase plane, and output plane positions on the output wavefront $w^{n}$ are analyzed with the beam propagation method [22] and numerical simulations. The distance settings of the input, phase, and output planes are shown in Figure 4a. Figure 4c shows the numerical simulation results of the effect of the displacement of the input plane on the wavefront of the output plane, and the step size in the displacement calculation is 0.01 mm. Figure 4d shows the numerical simulation results of the effect of the output plane displacement on the wavefront of the output plane, and the step size in the displacement calculation is 0.01 mm. Figure 4b shows the experimental results of the effect on the wavefront of the output plane in the optical axis direction when the output plane displacement is ±1 mm or ±2 mm. When the observation plane is shifted in the optical axis direction, the quality of the diffraction image is reduced, which leads to incorrect links in the output of the neuron nodes in the diffraction optical neural network. The holographic template that was designed for the experimental alignment of the optical diffraction neural network target classification device is shown in Figure 3e, and the pattern of the holographic mask in the output plane is shown in Figure 3d.

### 3.2. Robustness between Network Layers

**Figure 5:**
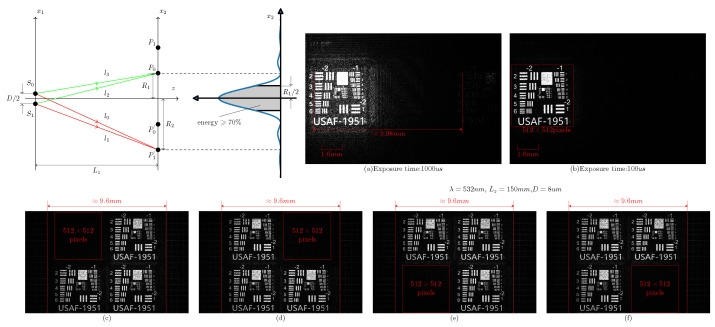
First- and second-order diffraction patterns.

The implementation of multilayer networks in blocks in a plane requires that the interference between blocks in different network layers be analyzed. Due to the independence of light propagation, there is no interlayer interference in the free propagation process; thus, the analysis of the interference between blocks in different network layers needs to consider only the distribution and energy of the first-order diffraction between different blocks in the same plane. As shown in Figure 5, there are two parallel planes, $x_{1}$ and $x_{2}$, in the direction of optical axis *z*, and there is a rectangular hole aperture of size *D* in plane $x_{1}$. $R_{1}$ is the zero-order diffraction half-width, and $|R_{2}-R_{1}|$ is the distance between the zero-order diffraction pattern and the first-order diffraction pattern. Figure 5a shows the zero-order and first-order diffraction patterns acquired by CMOS. The bright diffraction pattern on the left is the zero-order diffraction pattern, and the dark pattern on the right is the first-order diffraction pattern. The distance between the phase mask and the CMOS detector is 150 mm, the pixel size of the phase template is 8 um, and the laser wavelength is 532 nm. Thus, according to Equation (6), the distance between the zero-order and first-order diffraction patterns is approximately 9.98 mm, which is consistent with the experimental results.

(6) $$\begin{array}{cc} \; & |l_{2}-l_{3}|=\lambda /2\\ \; & R_{1}\approx \frac{\lambda L_{z}}{D}\;L_{Z},R_{1}>>D,\lambda \end{array}$$

According to Equation (6), to prevent first-order diffraction interference between blocks in different network layers, multiple regions in the zero-order diffraction range can be divided into blocks in the different network layers. Figure 5c–f shows the experimental result of dividing multiple regions in the zero-order diffraction range into blocks in the different network layers. The activation threshold (Min) of the function Equation (5) is set to be larger than the energy of the first-order diffraction pattern. As shown in Figure 5b, the first-order diffraction interference is prevented by reducing the CMOS exposure time. In addition to preventing first-order diffraction interference, the division of different network layer into blocks should consider the connectivity between neurons in the input plane, phase plane and output plane. The connectivity between neurons in the phase and output planes can be determined based on the distance $L_{Z}$ between the diffraction and output planes, the neuron size *D* in the phase and output planes, and the wavelength λ of the light source. When the neurons in the phase and output planes of the network layer are fully connected, the size of the phase and output planes *R* can be calculated with Equation (7):

(7) $$\begin{array}{cc} \; & R_{max}=\frac{\lambda L_{z}}{D}\;L_{Z},R>>D,\lambda \end{array}$$

### 3.3. Classification Experiments and Results

**Figure 6:**
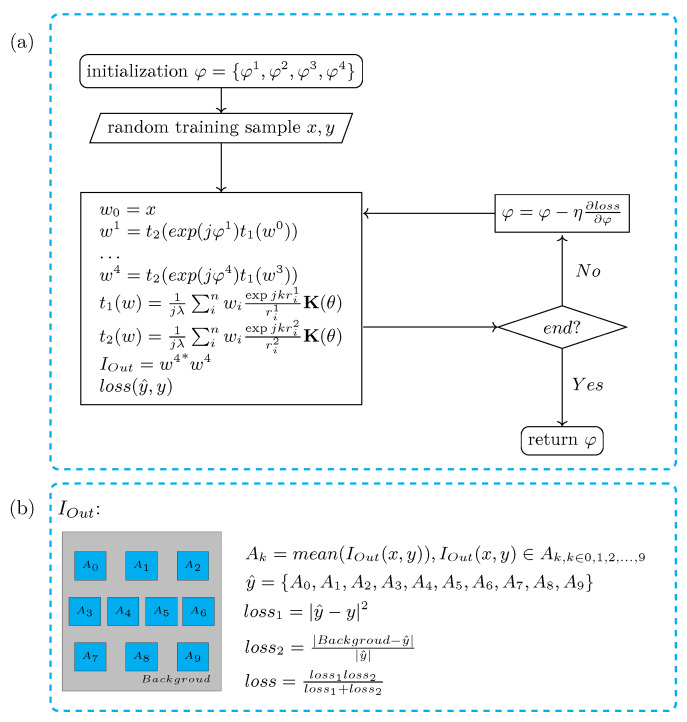
Training process and loss function. (**a**) Flow chart of ONN training. (**b**) Loss function. ($A_{0,\cdots ,9}$: The mean value of the light intensity in the cyan part of the picture; *background*: The mean value of the light intensity in the gray part of the picture).

**Figure 7:**
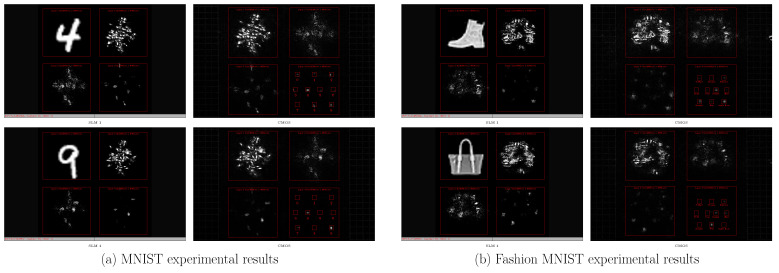
MNIST dataset and Fashion-MNIST dataset classifier.

The classification performance of the proposed partitionable and efficient multilayer diffractive optical neural network is validated with the Fashion-MNIST dataset [23] and the MNIST dataset [24]. The training set contains approximately 50,000 images, and the test set contains approximately 10,000 images. The network architecture of the four-layer network is shown in Figure 2d; the input plane, phase plane, and output plane in the fully connected layer all have sizes of 512×512, and the neuron size is 8×8 um. The network training process is shown in Figure 6a. The network classification output $\hat{y}=\left\{A_{0},A_{1},A_{2},A_{3},A_{4},A_{5},A_{6},A_{7},A_{8},A_{9}\right\}$ is the mean value of the light intensity in the ten regions in the output layer, and the ten cyan regions $A_{0,1,\cdots ,9}$ in Figure 6b indicate the divisions used in the classification experiments in this paper, where $A_{0,1,\cdots ,9}$ corresponds to ten different categories of outputs, and the correspondence is shown in Figure 7 for the output layer Layer4Out.

The loss function of the optical neural network in this paper is shown in Figure 7b. The smaller the value of $Loss_{1}$, the better the classification rate of the network. $Loss_{2}$ is the loss of the quality in the output layer of the classification network, which is designed to prevent stray light spots in the output layer. Figure 7a shows the data collected by SLM 1 at the input plane and the light field intensity distribution collected by CMOS at the output plane for the MNIST dataset classification experiment. Figure 7b shows the data collected by SLM 1 at the input plane and the light field intensity distribution collected by CMOS at the output plane for the Fashion-MNIST dataset classification experiment.

**Figure 8:**
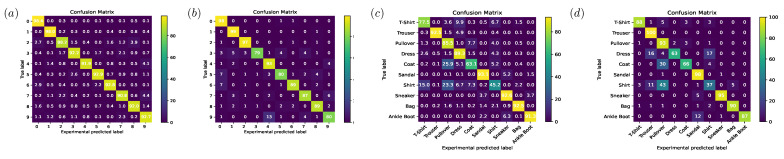
Confusion matrix of the MNIST [24] and Fashion-MNIST [23] test set classification results. (**a**) Digital simulation of the MNIST test set classification results. (**b**) Optical experiment of the MNIST test set classification results. (**c**) Digital simulation of the Fashion-MNIST test set classification results. (**d**) Optical experiment of Fashion-MNIST test set classification results.

Table 1 shows the classification accuracies of our proposed partitionable diffractive optical neural network compared with the state-of-the-art diffractive optical neural network. Figure 8a shows the confusion matrix of the simulation results for the MNIST test set. The test set includes 10 categories, with approximately 1000 images per category, and the classification accuracy is 93%. Figure 8b shows the confusion matrix of the results of the optical experiments on the MNIST test set. The dataset has 100 images per category, and the classification accuracy is 89.1%. Figure 8c shows the confusion matrix of the simulation results for the Fashion-MNIST test set. The test set includes 10 categories, with approximately 1000 images per category, and the classification accuracy is 82.9%. Figure 8d shows the confusion matrix of the results of the optical experiments on the Fashion-MNIST test set. The data include 1000 randomly selected images from the Fashion-MNIST test set, with approximately 100 images for each category, and the classification accuracy is 81.7%.

## 4. Discussion

### 4.1. Estimation of the Computational Speed of Multi-Layer Networks

**Figure 9:**
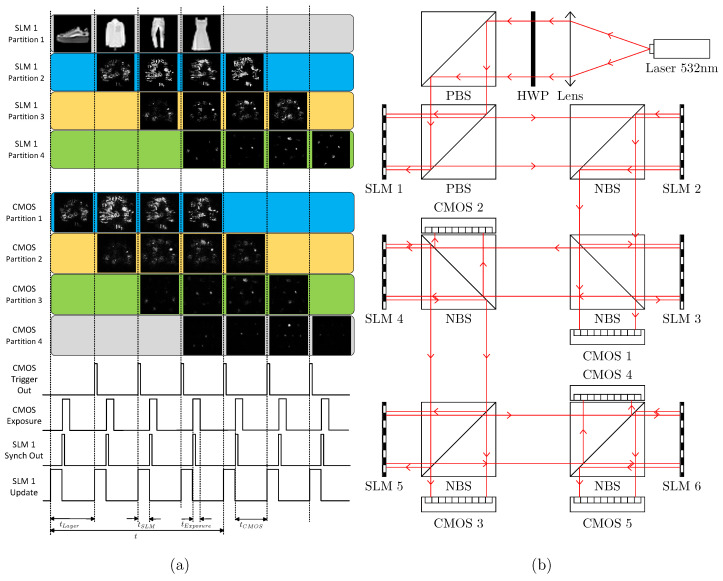
(**a**) Sequence diagram of partitionable multilayer (four layer) diffractive optical neural network. (**b**) Partitionable diffractive optical neural network config with higher number of layers.

To apply the proposed method, the output of the first partition of the CMOS sensor must be used as the input of the second partition of SLM1. Similarly, the output of the second partition of the CMOS sensor must be used as the input of the third partition of SLM1, and so on; therefore, we use a partitionable multilayer optical neural network refreshing strategy as shown in sequence diagram Figure 9a. Each cycle of the data update in each network layer of the partitionable multilayer optical neural network was programmed and triggered by software commands, with the output synchronization TTL signal of SLM 1 being used to trigger the exposure of the CMOS sensor and the readout TTL signal of the CMOS sensor being used to trigger the data update of SLM 1.

The specific details of the computational time consumption of our experimental diffractive optical neural network are shown in Figure 9a. The sequence diagram of four consecutive input images of the four-layer diffractive optical neural network is shown in Figure 9a. All partitions of SLM 1 are updated with data synchronously, where $t_{SLM}$ is the response time of SLM 1. When CMOS receives the synchronous trigger signal from SLM 1 to trigger all partitions on CMOS start to expose at the same time, the exposure time is $t_{Exposure}$, in our experiment $100\;\mu s\leq t_{Exposure}\leq 400\;\mu s$. $t_{CMOS}$ is the time required for CMOS to acquire a frame, and $t_{Layer}$ is the time for the diffractive optical neural network layer to refresh the data once. *t* is the computational delay of the diffractive optical neural network. In our experiments $t_{SLM}=16.7\;\mathrm{ms}$, $t_{CMOS}=46.3\;\mathrm{ms}$, $t_{Layer}=t_{SLM}+t_{CMOS}=63\;\mathrm{ms}$, $t=4*t_{Layer}=252\;\mathrm{ms}$.

For diffractive optical neural networks with higher number of layers, our proposed diffractive optical neural network structure should be suitably extended to avoid excessive network computation delay. If the optical path structure as shown in Figure 3 is still used, the computational delay of the N-layer network is $t_{Layer}\times N$, and a larger spatial light modulator and CMOS detector need to be replaced when N≥4 to achieve more partitions. The optical path structure of the diffractive optical neural network with 1 to 20 layers tunable is shown in Figure 9b. When the number of network layers for diffractive optical neural network calculation is 20, SLM 2 loads the network weights of layer 1+i×5, SLM 3 loads the network weights of layer 2+i×5, SLM 4 loads the network weights of layer 3+i×5, SLM 5 loads the network weights of layer 4+i×5, and SLM 6 loads the network weights of layer 5+i×5. CMOS 1-4 is turned off, and CMOS 5 is enabled. The input of the network is input from the first partition of SLM 1, which is output to the first partition of the CMOS 5 detector through the first partition of SLM 2-6 to complete the calculation of the network at layers 1 to 5. The output of the first partition of the CMOS 5 detector is used as input to the second partition of SLM 1, which is output to the second partition of the CMOS 5 detector through the second partition of SLM 2-6 in turn. The second partition of SLM 1 is used as input to the second partition of SLM 1. Similarly, the data of the second partition of CMOS 5 detector are used as the input of the third partition of SLM 1, and so on. The computation delay of the network at this point is $t=(t_{CMOS}+t_{SLM})\times 4$.

### 4.2. Limits of Partitionable Multilayer Diffractive Optical Neural Network

Partitioning on spatial light modulators and CMOS detectors to implement multilayer diffractive optical neural networks requires concern for the size of the partition. We tested diffractive optical neural networks with phase mask of different resolutions and phase mask of different pixel sizes by simulation experiments. Table 2 shows the training classification accuracy and testing classification accuracy of our simulated four-layer diffractive optical neural network for MNIST dataset with different resolution and different pixel size of phase mask. According to the results in Table 2, the classification accuracy of the network does not increase linearly with the number of phase plate pixels, and the pixels size of the phase mask also affects the classification performance of the diffractive optical neural network.Classification accuracy of MNIST dataset for partitionable diffractive optical neural networks with different resolutions and pixel sizes of phase mask. This can be explained by our experiments in Section 3.2, using the parameters of the phase mask in our experiments as an example: the pixel size is D=8μm and the resolution of phase mask is 512×512. The size of the phase mask is 4.096mm×4.096mm; the distance from the phase mask to the CMOS sensor is 150mm, and according to Equation (7), it can be calculated that more than 70% of the energy emitted from the 8μm×8μm sized point source on the phase mask is concentrated in the area with a diameter of 9.97mm. However, according to Rayleigh’s criterion, the resolution limit of the optical aperture of 4.096mm×4.096mm at a distance of 150mm is 1.22λ*f/D=16.8μm, λ=532nm, f=150mm, $D=4.096\;\mathrm{mm}*\sqrt{2}$; more than 70% of the energy is concentrated in the circle of radius 8.4μm, the activated pixel size range is 16.8 μm∼8.4 μm. As shown in Table 2, the phase mask resolution of 256×256 for a pixel size of 16μm and the phase mask resolution of 512×512 or 1024×1024 for a pixel size of 8μm satisfies our calculation results and the data in the table also show a high classification accuracy in training and testing.

## 5. Conclusions

In this paper, we propose a partitionable and efficient multilayer diffractive optical neural network architecture. This model addresses a disadvantage of the D2NN network, in which it is difficult to flexibly change the number of layers and the scale of the input data, by partitioning the optical diffractive devices in a multilayer network. The greatest advantage of partitioned multiplexing is that this method can improve the utilization of diffractive devices and the computational efficiency of the whole network while reducing the number of optical devices and the difficulty of assembling and adjusting the optical system. In addition to the above advantages, the network model achieves a classification performance similar to mainstream diffractive optical neural networks. Because the framework is not limited to the visible spectrum and can easily be extended to other spectra, this system has great application value. 

## Figures and Tables

**Table 1:** Accuracies of the MNIST and Fashion-MNIST dataset classifiers.

	Method	Digital Simulation	Optical Experiment	Taining Time	Layers
MNIST	purposed	93% (10,000)	89.1% (1000)	4 h	4
	D2NN (Thz) [11]	91.7% (10,000)	88% (50)	8 h	5
	D2NN (632 nm) [15]	91.57% (10,000)	84% (50)	20 h	5
Fashion	purposed	83.9% (10,000)	81.7% (1000)	4 h	4
	D2NN (Thz) [11]	81.1% (10,000)	90% (50)	8 h	5
	D2NN (632 nm) [15]	-	-	-	-

**Table 2:** Classification accuracy of partitionable diffractive optical neural networks with different resolutions and pixel sizes of phase mask.

Mask Size	Pixel Size	Epoch	Train	Test	Train (Nonlinear)	Test (Nonlinear)
64 × 64	8μm	100	19.2±0.5%	19.0±0.1%	11.2±0.5%	11.0±0.5%
64 × 64	16μm	100	58.0±0.5%	57.4±0.5%	52.5±0.5%	52.0±0.5%
64 × 64	24μm	100	64.5±0.5%	64.5±0.5%	65.5±0.5%	64.8±0.5%
64 × 64	32μm	100	69.8±0.5%	69.0±0.5%	70.7±0.5%	70.5±0.5%
128 × 128	8μm	100	58.5±0.5%	58.0±0.5%	49.8±0.5%	49.3±0.5%
128 × 128	16μm	100	78.2±0.5%	76.1±0.5%	74.2±0.5%	76.5±0.5%
128 × 128	24μm	100	81.6±0.5%	80.4±0.5%	84.1±0.5%	85.1±0.5%
128 × 128	32μm	100	78.3±0.5%	77.1±0.5%	85.1±0.5%	85.5±0.5%
256 × 256	8μm	100	75.6±0.5%	75.5±0.5%	74.5±0.5%	74.4±0.5%
256 × 256	16μm	100	86.9±0.5%	86.5±0.5%	90.2±0.5%	90.1±0.5%
256 × 256	24μm	100	81.1±0.5%	81.5±0.5%	88.1±0.5%	88.0±0.5%
256 × 256	32μm	100	78.2±0.5%	78.1±0.5%	78.2±0.5%	78.0±0.5%
512 × 512	8μm	100	87.6±0.5%	87.2±0.5%	92.3±0.5%	92.0±0.5%
512 × 512	16μm	100	84.3±0.5%	84.0±0.5%	89.5±0.5%	89.2±0.5%
512 × 512	24μm	100	76.8±0.5%	76.0±0.5%	76.1±0.5%	75.7±0.5%
512 × 512	32μm	100	75.9±0.5%	68.0±0.5%	65.5±0.5%	64.5±0.5%
1024 × 1024	8μm	100	87.5±0.5%	86.2±0.5%	92.0±0.5%	90.8±0.5%
1024 × 1024	16μm	100	76.5±0.5%	74.5±0.5%	78.0±0.5%	76.5±0.5%
1024 × 1024	24μm	100	63.0±0.5%	62.0±0.5%	61.0±0.5%	60.2±0.5%
1024 × 1024	32μm	100	47.0±0.5%	45.0±0.5%	48.0±0.5%	46.5±0.5%

## Data Availability

Not applicable.
